# Chinese olive extract ameliorates hepatic lipid accumulation *in vitro* and *in vivo* by regulating lipid metabolism

**DOI:** 10.1038/s41598-018-19553-1

**Published:** 2018-01-18

**Authors:** Yu-Te Yeh, Yan-Yu Cho, Shu-Chen Hsieh, An-Na Chiang

**Affiliations:** 1 Institute of Food Science and Technology, National Taiwan University, Taipei, Taiwan; 2 Institute of Biochemistry and Molecular Biology, National Yang-Ming University, Taipei, Taiwan

## Abstract

Chinese olive contains plenty of polyphenols, which possess a wide range of biological actions. In this study, we aimed to investigate the role of the ethyl acetate fraction of Chinese olive fruit extract (CO-EtOAc) in the modulation of lipid accumulation *in vitro* and *in vivo*. In cellular studies, CO-EtOAc attenuated oleic acid-induced lipid accumulation; we then elucidated the molecular mechanisms of CO-EtOAc in FL83B mouse hepatocytes. CO-EtOAc suppressed the mRNA levels of fatty acid transporter genes (*CD36* and *FABP*) and lipogenesis genes (*SREBP-1c*, *FAS*, and *ACC1*), but upregulated genes that govern lipolysis (*HSL*) and lipid oxidation (*PPARα*, *CPT-1*, and *ACOX*). Moreover, CO-EtOAc increased the protein expression of phosphorylated AMPK, ACC1, CPT-1, and PPARα, but downregulated the expression of mature SREBP-1c and FAS. AMPK plays an essential role in CO-EtOAc-mediated amelioration of lipid accumulation. Furthermore, we confirmed that CO-EtOAc significantly inhibited body weight gain, epididymal adipose tissue weight, and hepatic lipid accumulation via regulation of the expression of fatty acid transporter, lipogenesis, and fatty acid oxidation genes and proteins in C57BL/6 mice fed a 60% high-fat diet. Therefore, Chinese olive fruits may have the potential to improve the metabolic abnormalities associated with fatty liver under high fat challenge.

## Introduction

Chinese medicinal herbs and phytochemicals are known to play a major role in the discovery of new therapeutic agents and have received much attention as sources of bioactive substances including anti-oxidative, hypoglycaemic and hypolipidemic agents^1,2^. Considering the finding that hydroxytyrosol, a polyphenol mainly present in extra virgin olive oil, provides diverse benefits for the prevention of metabolic disorder^3^. We suggest that the extract from olive fruits could offer strong benefit for human health. Chinese olive (*Canarium album* L.) is a tropical and semi-tropical plant that is widely cultivated in Taiwan, Southeast China, and other regions of Asia. Different from the species of *Olea europaea* L. produced from Mediterranean Europe, Chinese olive fruits contain many bioactive components with anti-oxidative, anti-inflammatory, and hepatoprotective activities^4^. The prevalence of non-alcoholic fatty liver disease (NAFLD) in the general population is estimated to be 20–30% in western countries and 5–18% in Asia; this figure is increasing over time^5^. Currently, there is no effective drug therapy for NAFLD, great efforts are focused on finding novel dietary ingredients, interventions in lifestyle or behavioral therapies for prevention of the progression of fatty liver to nonalcoholic steatohepatitis^6^. Some natural products from herbal extracts can effectively offer antihyperlipidemic and hepatoprotective effects against NAFLD^7^.

Previous study has demonstrated that HFD elicits long chain polyunsaturated fatty acids depletion via the reduction in activity of hepatic Δ5- and Δ6- desaturases, which promotes a pro-steatotic effect and oxidative stress in the liver^8^. Moreover, cultured hepatocytes under high concentrations of either palmitate or oleic acid (OA) can also lead to differential lipotoxic effects^9^. Lipid accumulation in the liver can be responsible for changes in lipid transport, *de novo* lipogenesis (DNL), lipolysis, and fatty acid oxidation, consequently resulting in dyslipidemia^10^. Free fatty acids (FFAs) are taken into hepatic cells by passive diffusion or through protein-mediated transport mechanisms, including fatty acid translocase CD36^11^, fatty acids transporters (FATs) such as FATP2 and FATP5^12,13^, and fatty acid binding protein (FABP)^14^. However, hepatic CD36 expression is greater in fatty liver disease model rodents, which subsequently increases FFA uptake^15,16^. Clinical studies also demonstrate that an upregulation of hepatic CD36 and FABP was associated with hepatic fatty acid infiltration in patients with NAFLD^17,18^. Excess FFAs are rapidly esterified within hepatocytes to form diglycerides (DG)^19^ and triglycerides (TG)^20^. Thus, a significant increase in hepatic DG, TG, and total lipid content is observed in patients with NAFLD and non-alcoholic steatohepatitis (NASH)^21^. DNL mainly takes place in adipose tissue and the liver mediated by enzymes including acetyl-CoA carboxylase 1 (ACC1) and fatty acid synthase (FAS)^22^. ACC1 catalyses the carboxylation of acetyl-CoA, producing malonyl-CoA, which can be used by FAS for fatty acid biosynthesis^23^. FAS catalyses the last step in fatty acid biosynthesis, which is believed to be the major determinant to modulate DNL in the liver^24^. Sterol regulatory element binding proteins (SREBPs) are synthesized as inactive, ER membrane-bound precursors^25^. Through a proteolytic process, the NH_2_-terminal of the precursor SREBP is released and the mature SREBP translocates to the nucleus. Mature, nuclear SREBP subsequently activates transcription by binding sterol regulatory elements in promoters of the target genes. SREBP-1c is a transcription factor involved in the transcriptional activation of genes encoding FAS and ACC1 in lipogenesis. Previous studies have shown that dysregulation of SREBP-1c results in pathogenesis including hepatic steatosis, dyslipidaemia, and type 2 diabetes^26,27^.

Induction of lipolysis pathways in liver is another way to contribute to the prevention of hepatic steatosis^28^. During TG degradation (lipolysis), adipose triglyceride lipase (ATGL) initiates the cleavage of a TG to a diacylglycerol (DG) plus a fatty acid (FA)^29^. Hormone-sensitive lipase (HSL) mediates hydrolysis of DG to a monoacylglycerol (MG) plus a FA^30^ and monoglyceride lipase (MGL) hydrolyzes MG to glycerol and fatty acids^31^. Understanding the regulation of these enzymes that control lipolysis would be likely the essential targets for contribution to lipid metabolism. Another mechanism that has been postulated to contribute to lipid disorders is defective fatty acid oxidation. Acyl-CoA oxidase (ACOX) and carnitine palmitoyltransferase-1 (CPT-1) are the two enzymes responsible for the fatty acid oxidation pathways, whereas ACOX initiates the process of long-chain fatty acid oxidation and CPT-1 catalyses the rate-limiting step of fatty acid β-oxidation^32^. Peroxisome proliferator-activated receptor alpha (PPARα) is expressed predominantly in tissues that have a high level of fatty acid catabolism, such as liver, heart, and muscle^33^. Furthermore, activation of PPARα could ameliorate hepatic insulin resistance and protect the liver from NAFLD and NASH by promoting the target genes related to fatty acid oxidation^34,35^. Perturbation of the expression of these lipid regulatory enzymes or transcription factors could be detrimental to hepatic lipid homeostasis.

Emerging evidence suggests that metabolic abnormalities in fatty liver may result from the dysregulation of adenosine-monophosphate-activated protein kinase (AMPK) phosphorylation. AMPK is an energy sensor that regulates glucose and lipid metabolism, and could be an attractive target for the treatment of hepatic disorders^36^. Calcium-calmodulin/dependent protein kinase beta (CaMKKβ) and liver kinase B1 (LKB1) are the two master enzymes catalyzing AMPK phosphorylation^37,38^. Activation of AMPK has the potential to switch cells from an anabolic to a catabolic state, initiating an ATP-producing pathway, increasing fatty acid oxidation, and inhibiting hepatic lipogenesis^39,40^. AMPK also modulates SREBP-1c and PPARα, which play essential roles in the regulation of gene products responsible for the synthesis of cholesterol, fatty acids, and triglycerides in the liver^41,42^. The effects of AMPK activation provides rationale for the development of new pharmacological therapies and nutritional supplements to reverse metabolic disorders^43,44^.

Few studies have been performed on the effect of Chinese olive fruit and its bioactive compounds on the prevention of lipid accumulation *in vivo* and *in vitro*. The objective of this study was to investigate whether CO-EtOAc modulates lipid accumulation in FL83B hepatocytes and to examine its influence on genes and proteins involved in the regulation of fatty acid transport, lipogenesis, and lipid oxidation. Importantly, observations in the cell model have been verified in the livers of animals fed a high fat diet, and the involvement of AMPK in CO-EtOAc mediated reduction of lipid accumulation in hepatocytes was also confirmed.

## Results

### CO-EtOAc decreases lipid accumulation in OA-treated FL83B hepatocytes

**Figure 1:**
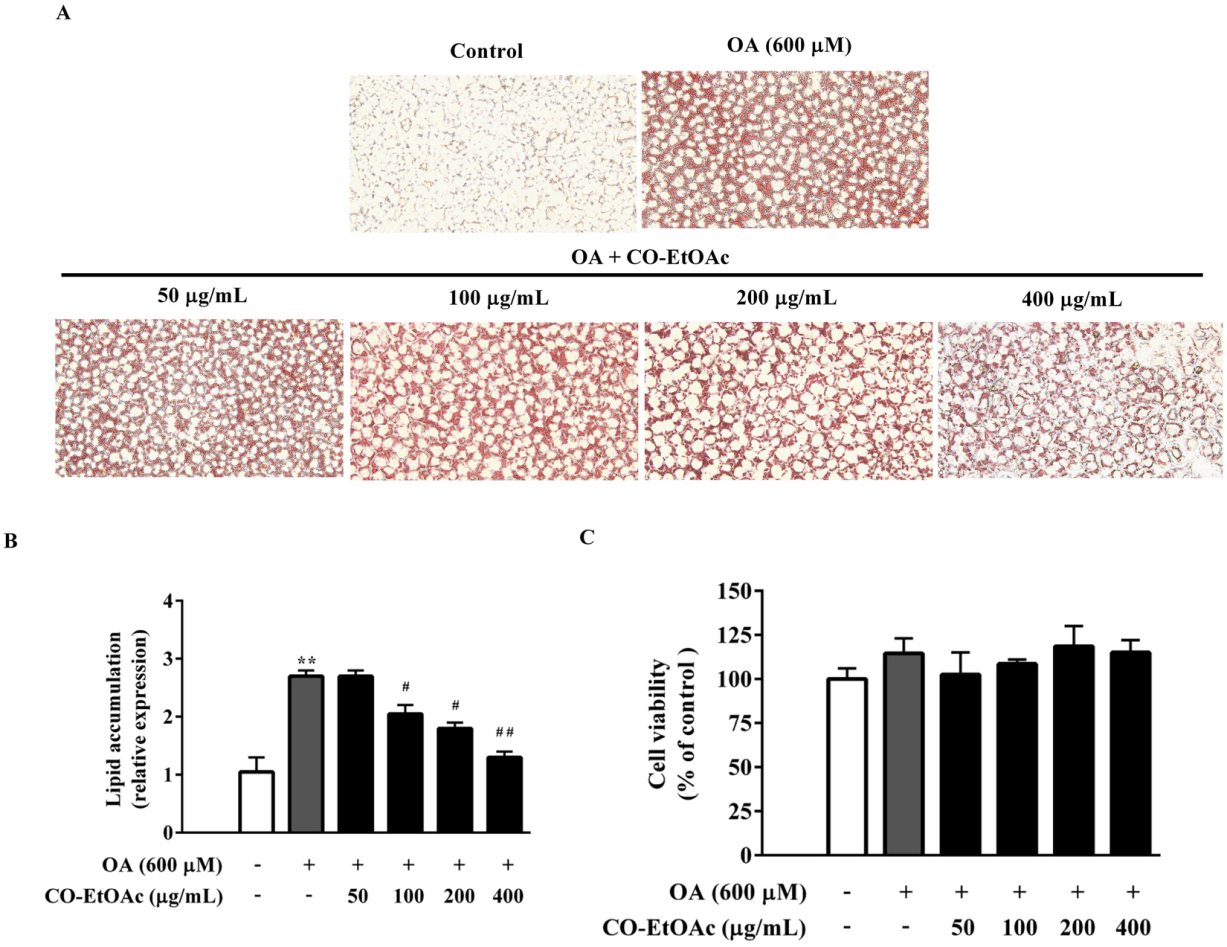
CO-EtOAc decreases oleic acid (OA)-induced lipid accumulation in FL83B cells. (**A**) Cells were exposed to 600 μM OA. After 3 h of this treatment, different concentrations (50, 100, 200, and 400 μg/mL) of Chinese olive fruit extract of ethyl acetate fractions (CO-EtOAc) were added for another 21 h, while a control group was treated with the same concentration of DMSO. Intracellular lipid accumulation was determined by Oil Red O assay. The Oil Red O stained lipid droplets were visualized by light microscope and photographed with a digital camera at 200X magnification. (**B**) Quantified intracellular fat drops were extracted using 100% isopropanol and measured using a microplate reader at 490 nm. The histogram shows the relative fold change compared with the control group (set as 1) (**C**) Cell viability was determined by MTT assay. All results are presented as the mean ± SEM of three independent experiments. Values are expressed as a percentage of the control group, which is set as 100%. **p < 0.01 versus the DMSO control group; ^#^*p* < 0.05, ^##^*p* < 0.01 versus the OA group.

To investigate the effect of CO-EtOAc on hepatic lipid accumulation, we established a cellular model of FL83B mouse hepatocytes. As shown in Fig. 1A, the addition of 600 μM oleic acid for 24 h led to an increase in cellular lipid accumulation, as assessed using Oil red O staining. However, co-treatment with 50–400 μg/mL CO-EtOAc for 21 h exhibited a significant decrease in lipid accumulation in a concentration-dependent manner (Fig. 1B). To exclude the possibility that the suppressive effect is due to a decreased cell number, we examined the effect of CO-EtOAc on cell viability at various concentrations. The results show that CO-EtOAc exhibited no toxic effect at the indicated dosages (Fig. 1C).

### CO-EtOAc reduces the production of reactive oxygen species and thiobarbituric acid reactive substances in OA-treated FL83B hepatocytes

**Figure 2:**
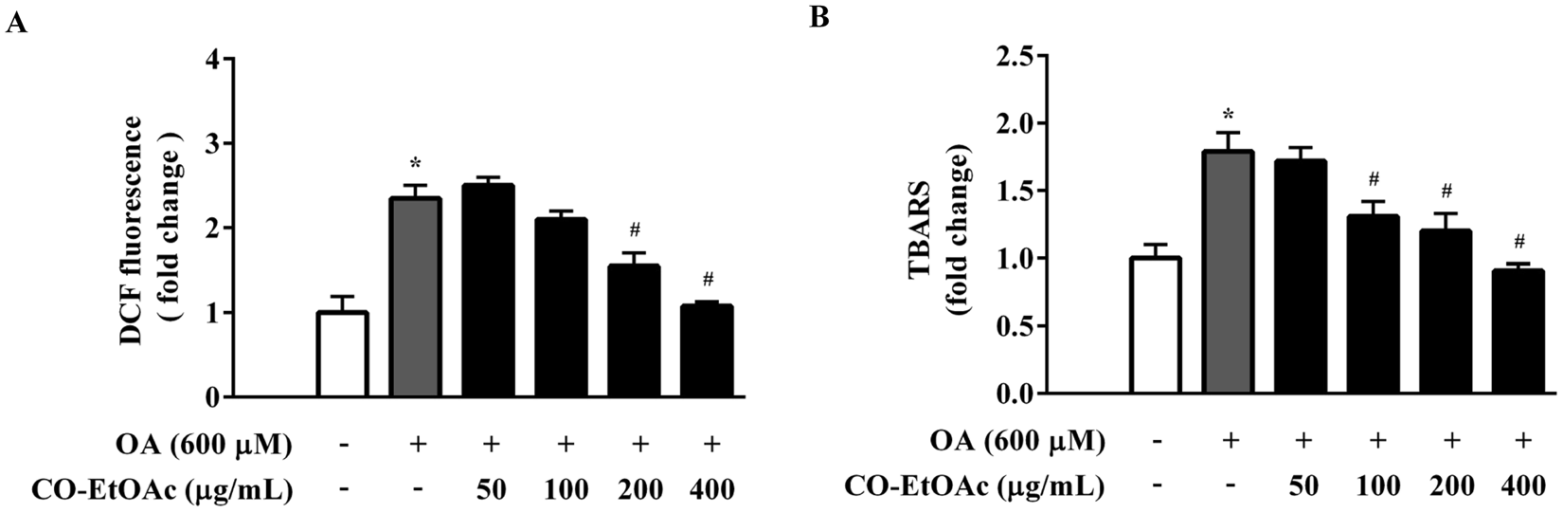
CO-EtOAc reduces oleic acid (OA)-induced ROS production and TBARS levels in FL83B hepatocytes. (**A**) Cells were pretreated with 600 μM OA for 3 h followed by treatment in the presence or absence of different concentrations (50–400 μg/mL) of CO-EtOAc for another 21 h. Intracellular ROS generation was analysed with 2′,7′-dichlorodihydrofluorescein (DCF) using a fluorescence reader at an excitation wavelength of 485 nm and an emission wavelength of 535 nm. (**B**) MDA equivalents were determined using the thiobarbituric acid-reactive substances (TBARS) assay. All results are presented as the mean ± SEM of three independent experiments. Values are expressed as relative fold change compared with the control group, which is set as 1. *p < 0.05 versus the DMSO control group; ^#^*p* < 0.05 versus the OA group.

Since excess fat accumulation contributes to oxidative stress, which is the cause of many chronic diseases, we further investigated the effect of CO-EtOAc on the production of reactive oxygen species (ROS) and lipid peroxidation. We treated FL83B hepatocytes with 600 μM OA for 3 h, followed by co-treatment with different dosages of CO-EtOAc for another 21 h. OA increased intracellular ROS levels by 2.6 fold when compared with the control group; however, the increase was suppressed by CO-EtOAc treatment in a dose-dependent manner. At the highest dose of CO-EtOAc treatment, the ROS level was reduced to near control level (Fig. 2A). We also determined the effect of CO-EtOAc on thiobarbituric acid reactive substance (TBARS) levels. As shown in Fig. 2B, CO-EtOAc inhibited the OA-induced TBARS levels in FL83B cells, reaching a level comparable to the control level. These findings indicate that CO-EtOAc reduces OA-induced oxidative stress.

### CO-EtOAc regulates the expression of genes involved in fatty acid transport, *de novo* lipogenesis, lipolysis, and lipid oxidation in OA-treated hepatocytes

**Figure 3:**
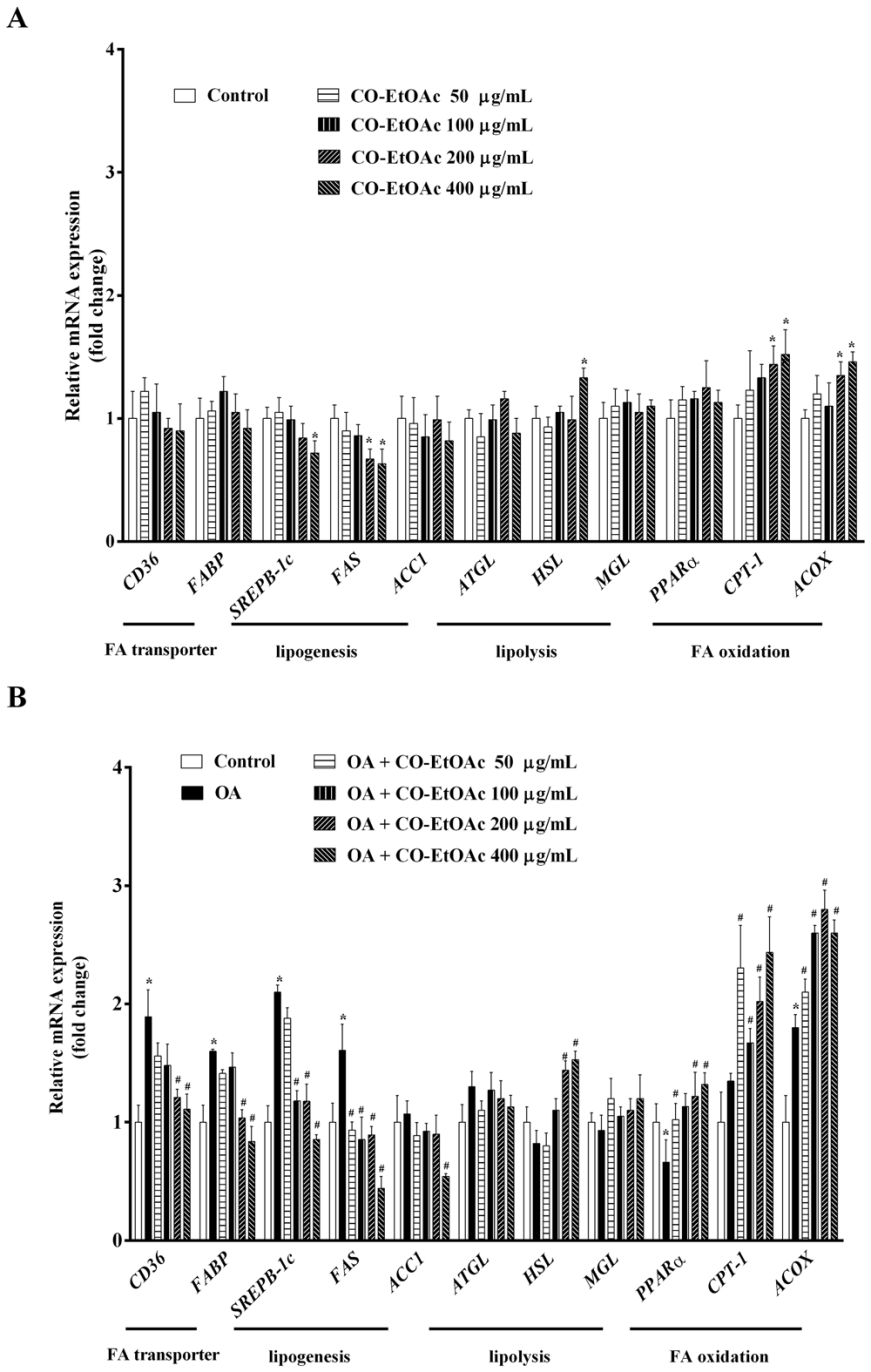
CO-EtOAc regulates the expression of lipid metabolism-related genes in FL83B hepatocytes. Total RNAs were extracted using Trizol reagent and mRNA was measured using qRT-PCR. Relative mRNA levels were normalized to the housekeeping gene, r18S. The fold changes relative to the control were calculated using the ΔΔCT method for mRNA expression levels of fatty acid transporter genes, *CD36* and *FABP*; lipogenic genes: *SREBP-1c*, *FAS*, and *ACC1*; fatty acid oxidation genes: *PPAR-α, CPT-1*, and *ACOX*. All experimental groups had been treated with the same concentration of DMSO. (**A**) Cells were incubated with different concentrations (50–400 μg/mL) of CO-EtOAc. (**B**) Cells were incubated with 600 μM OA in the presence or absence of different concentrations (50–400 μg/mL) of CO-EtOAc. All results are presented as the mean ± SEM of three independent experiments. Values are expressed as relative fold change compared with the control group, which is set as 1. *p < 0.05 versus the DMSO control group; ^#^*p* < 0.05 versus the OA group.

To address the molecular mechanism underlying the inhibitory effect of CO-EtOAc on lipid accumulation in FL83B hepatocytes, we examined the effects of CO-EtOAc on the expression of genes associated with lipid metabolism. As shown in Fig. 3A, we found that CO-EtOAc treatment at both 200 μg/mL and 400 μg/mL for 24 h significantly increased the expression levels of genes related to lipid oxidation and lipolysis, but decreased that of genes associated with lipogenesis; no effect on the mRNA expression of fatty acid transporters was observed. Next, we examined whether CO-EtOAc affected the expression of genes that govern membrane-associated fatty acid transport, *de novo* lipogenesis, and lipid oxidation in FL83B hepatocytes treated with OA. As shown in Fig. 3B, CO-EtOAc suppressed the OA-induced increase in mRNA levels of fatty acid transporters such as *CD36* and *FABP* genes. We also analysed the mRNA levels of the lipogenic proteins SREBP1-c, FAS, and ACC1. As expected, OA treatment led to a 2.1-fold and 1.6-fold increase in *SREBP-1c* and *FAS* mRNA levels, respectively, and this increase was significantly inhibited by CO-EtOAc. Finally, CO-EtOAc up-regulated the mRNA levels of genes involved in lipolysis (*HSL*) and lipid oxidation, such as *PPARα*, *CPT-1*, and *ACOX*. These findings show that CO-EtOAc exhibits a broad range of biological actions, which may include the modulation of fatty acid transport, *de novo* lipogenesis, and lipid oxidation.

### CO-EtOAc regulates the expression of lipid metabolism-related proteins in OA-treated FL83B hepatocytes

**Figure 4:**
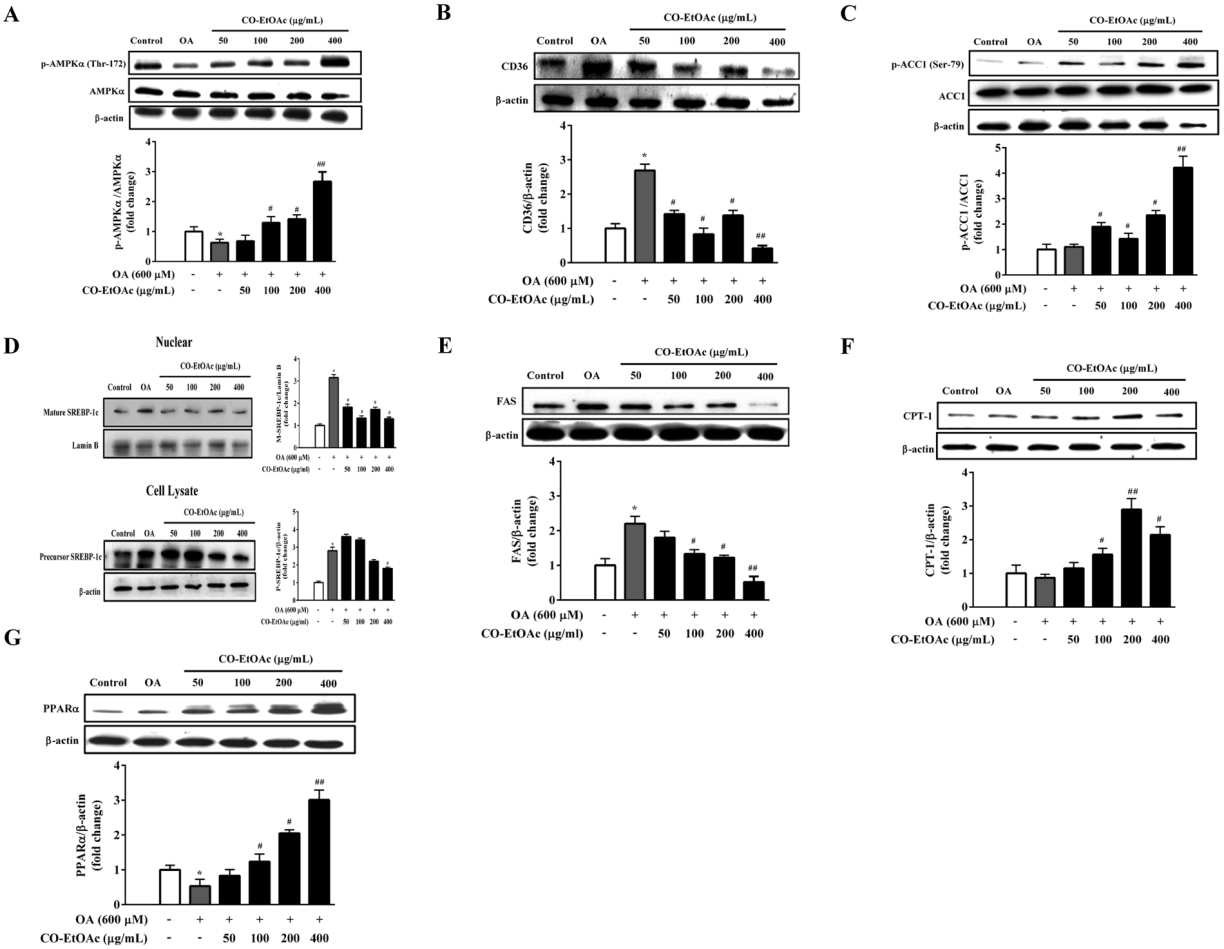
CO-EtOAc regulates the expression of lipid metabolism-related proteins in FL83B hepatocytes. Cells were pretreated with 600 μM OA for 3 h and then treated with different concentrations (50–400 μg/mL) of CO-EtOAc for another 21 h. All experimental groups and the control group were exposed to the same concentration of DMSO. Total proteins were extracted from whole cell lysates and then subjected to SDS-PAGE and immunoblotting with the indicated antibodies. The levels of protein expression were measured by western blot analysis. The intensity of the protein band was normalized against the internal control β-actin. Histograms depict the quantitative analysis of the results and the value for cells treated with BSA alone was set as 1. Representative bands of (**A**) phosphorylated and total AMPKα (**B**) CD36 (**C**) phosphorylated and total ACC1 (**D**) mature and precursor SREBP-1c (**E**) FAS (**F**) CPT-1 (**G**) PPARα were detected by western blot. All results are expressed as the mean ± SEM of three independent experiments. Values are express as relative fold change compared with the DMSO control group, which is set as 1. *p < 0.05 versus the DMSO control group; ^#^*p* < 0.05, ^##^*p* < 0.01 versus the OA group.

AMPK is a metabolic sensor that normalizes lipid, glucose, and energy imbalances^37^. Western blot analysis indicated that 600 μM OA suppressed the phosphorylation of AMPKα at Thr-172 in FL83B cells, whereas CO-EtOAc significantly enhanced phosphorylated AMPK levels in a dose-dependent manner (Fig. 4A). The maximum effect on the activation of phosphorylated AMPK was a 3.2-fold increase (*p* < 0.01 versus OA) at 400 μg/mL CO-EtOAc. Consistent with the role of AMPK in the regulation of lipid metabolism, we observed that 400 μg/mL of CO-EtOAc significantly decreased CD36 protein expression (*p* < 0.01 versus OA) by 4.2 fold (Fig. 4B) and enhanced the protein levels of phosphorylated ACC1, a downstream substrate of AMPK by 3.7 fold (*p* < 0.01 versus OA, Fig. 4C). To investigate whether expression of SREBP-1c is regulated by CO-EtOAc, mature and precursor forms of SREBP-1c were determined in the nuclear extracts and cell lysate, respectively, in the OA-treated FL83B hepatocytes. The mature form of SREBP-1c was significantly lower in the nuclear extract of CO-EtOA-treated cells (*p* < 0.01 versus OA, Fig. 4D), whereas the precursor form of SREBP-1c was not significantly changed in the cell lysate. Cells treated with CO-EtOAc also exhibited a strong dose-dependent downregulation of another AMPK downstream lipogenic enzyme, FAS, compared to the OA-treated group (Fig. 4E). In addition to the impact of CO-EtOAc on lipogenic protein expression, the effect on the regulation of proteins involved in lipid oxidation was also examined. We found that the expression of CPT-1 and PPARα significantly increased in the CO-EtOAc group (Fig. 4F and G), suggesting that CO-EtOAc may promote hepatic lipid oxidation under OA administration. These results indicate that CO-EtOAc decreases lipogenesis and increases lipid oxidation in OA-treated FL83B hepatocytes.

### The regulation of CO-EtOAc on lipid metabolism is dependent on AMPK in OA-treated FL83B hepatocytes

**Figure 5:**
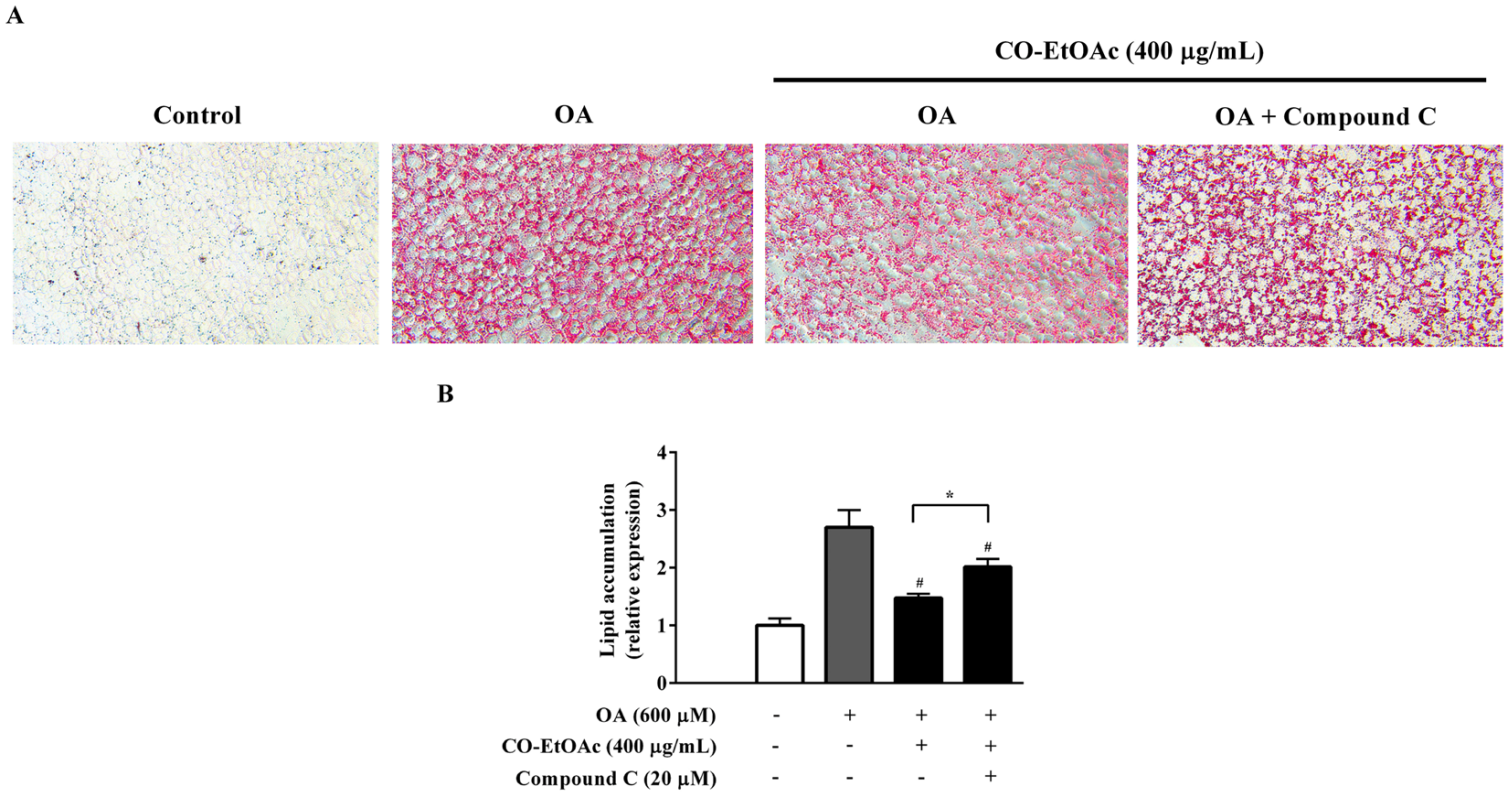
The effect of CO-EtOAc on lipid accumulation with OA is dependent on AMPK in FL83B hepatocytes. (**A**) Cells were pretreated with 20 µM compound C (an AMPK inhibitor) for 1 h followed by treatment with 600 μM OA for 3 h and then treated with 400 μg/mL of CO-EtOAc for another 21 h. All experimental groups and control groups were exposed to the same concentration of DMSO. Intracellular lipid accumulation was determined by Oil Red O assay. The Oil Red O stained lipid droplets were visualized by light microscope and photographed with a digital camera at 200X magnification. (**B**) To quantitate Oil Red O content levels, isopropanol was added to each sample shaken at room temperature for 5 min, and samples were measured using a microplate reader at 490 nm. All results are presented as the mean ± SEM of three independent experiments. Values are expressed as a percentage change compared with the control group, which is set as 100%. *p < 0.05 indicated the statistics between OA+ CO-EtOAc and OA+ CO-EtOAc+ compound C group; ^#^*p* < 0.05 versus the OA group.

In order to confirm the relationship between AMPK activation and the suppression of lipid accumulation in response to treatment with CO-EtOAc, we inhibited AMPKα activity using its chemical inhibitor and then detected the lipid contents of the FL83B hepatocyte. The cells were pretreated with 20 µM compound C (an AMPK inhibitor) for 1 h, followed by treatment with 600 μM OA for 3 h and then co-treatment with 400 μg/mL of CO-EtOAc for another 21 h. As revealed in Fig. 5A and B, compound C treatment significantly reversed the CO-EtOAc-mediated suppression of the lipid accumulation caused by OA.

### CO-EtOAc improves biochemical characteristics and ameliorates hepatic lipid accumulation via inhibition of lipogenesis, fatty acid transport, and promotion of fatty acid oxidation in high-fat treated C57BL/6 mice

**Figure 6:**
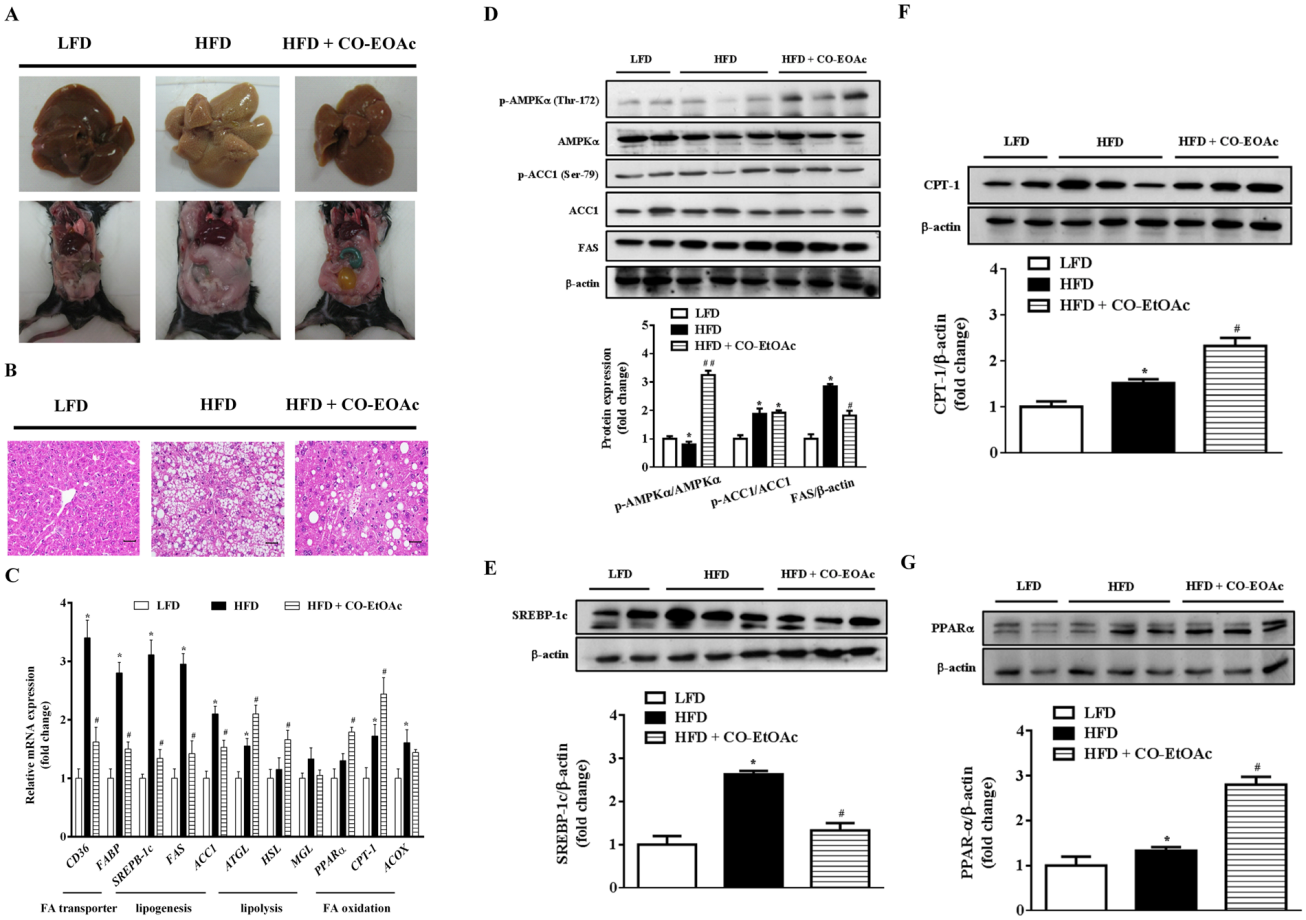
CO-EtOAc attenuates hepatic lipid accumulation in C57BL/6 mice treated with 60% high-fat diet (HFD). C57BL/6 mice were fed a high fat diet (HFD) for 21 weeks and treated with CO-EtOAc in the last 4 weeks. The control group was administered a low-fat diet (LFD). At the end of the experiment, all mice were killed by CO_2_ anaesthesia. The liver samples were fixed in 10% formalin for 24 h, embedded in paraffin, sectioned, and stained with haematoxylin and eosin (H&E). (**A**) Representative photographs of the liver appearance (upper) and the abdominal fat (lower) of mice among different groups are shown. (**B**) Histological features of the lipid accumulation in the livers of C57BL/6 mice. Representative photographs are shown with 100X magnification. All scale bars indicate 50 μm. (**C**) Total RNAs were extracted using Trizol reagent and mRNA was measured using qRT-PCR. Relative mRNA levels were normalized to the housekeeping gene, r18S. The fold changes relative to the control were calculated using ΔΔCT method for mRNA expression levels of fatty acid transporter genes, *CD36* and *FABP*; lipogenic genes: *SREBP-1c*, *FAS*, and *ACC1*; fatty acid oxidation genes: *PPARα, CPT-1*, and *ACOX*. The levels of protein expression of (**D**) phosphorylated and total AMPKα, phosphorylated and total ACC1, and FAS (**E**) SREBP-1c (**F**) CPT-1 (**G**) PPAR-α were measured by western blot analysis. The intensity of the protein band was normalized against the internal control β-actin. Histograms depict the quantitative analysis of the results and the value for the control group was set as 1. All results are expressed as the mean ± SEM of three independent experiments. *p < 0.05 versus the LFD group; ^#^*p* < 0.05, ^##^*p* < 0.01 versus the HFD group.

With the incentive to elucidate the physiological significance of CO-EtOAc on fatty liver, we established an animal model of emerging fatty liver via feeding C57BL/6 mice a 60% high-fat diet (HFD). After 21 weeks of HFD feeding, with or without supplement of CO-EtOAc in the last 4 weeks, all the mice exhibited normal physical activity and appearance. Daily food intake was similar among the three groups. Compared with mice treated with low-fat diet (LFD), HFD-fed mice had a significant increase in body and epididymal adipose tissue weight, hepatic levels of total cholesterol and triglyceride, and blood levels of glucose, insulin and triglyceride; administration of CO-EtOAc attenuated these changes (Table 1). Furthermore, changes in hepatic appearance and increases in abdominal fat accumulation caused by HFD were ameliorated after CO-EtOAc administration (Fig. 6A). Liver pathology was also examined by histological analyses. As shown in Fig. 6B, lipid droplets were diffusely present in the livers of mice fed 60% HFD, but significantly decreased in the CO-EtOAc-fed mice. In order to confirm the mechanism by which CO-EtOAc can attenuate HFD-induced lipid accumulation as shown in the cellular model, we investigated genes involved in fatty acid transport, *de novo* lipogenesis and lipid oxidation. As shown in Fig. 6C, we found that HFD-induced elevation of hepatic *CD36* and *FABP* gene expression levels was attenuated by CO-EtOAc administration, thereby leading to a decreased availability of fatty acids for biosynthesis and preventing lipid accumulation. Furthermore, HFD elevated hepatic *SREBP-1c*, *FAS*, and *ACC1* gene expression levels compared to that in mice fed with LFD. CO-EtOAc supplementation markedly suppressed the expression of these genes, indicating that CO-EtOAc had a greater effect on the downregulation of lipogenic gene expression. We also observed that CO-EtOAc was able to significantly upregulate the expression of *PPARα* and *CPT-1*, which may play essential roles in the regulation of gene products responsible for fatty acid oxidation. Consistently, hepatic protein levels of CD36, FABP, SREBP-1c, and FAS were all elevated in the HFD-treated group, however, CO-EtOAc reversed the induction (Fig. 6D,E). Furthermore, CO-EtOAc supplementation markedly enhanced the protein expression of phosphorylated AMPK, CPT-1 and PPARα compared to the HFD-treated group (Fig. 6F,G). HFD-induced elevation of the hepatic protein expression levels of FAS and SREBP-1 was also significantly attenuated by CO-EtOAc administration, thereby leading to a decreased lipid biosynthesis and prevention of lipid accumulation in mouse liver.

**Table 1:** Biochemical characteristics in C57BL/6 mice.

Measurements	LFD	HFD	HFD + CO-EtOAc
Food intake (g/d)	7.2 ± 0.18	8.1 ± 0.22	7.6 ± 0.53
Body weight			
Wk-0 body weight (g)	16.65 ± 0.40	15.42 ± 0.51	17.63 ± 0.68
Wk-17 body weight (g)	23.42 ± 0.33	37.36 ± 2.15^*^	36.92 ± 1.37^*^
Wk-21 body weight (g)	25.61 ± 0.24	39.52 ± 3.22^*^	35.36 ± 2.99
Body weight gain (g)	8.96 ± 0.16	24.10 ± 2.71^*^	17.93 ± 2.31^#^
EAT weight (g)	0.40 ± 0.05	2.03 ± 0.18^*^	1.28 ± 0.21^#^
Blood glucose (mg/dL)	136.22 ± 5.36	209.35 ± 10.26^*^	1 92.38 ± 20.64^#^
Serum			
Insulin (ng/mL)	0.45 ± 0.08	1.33 ± 0.86^*^	1.27 ± 0.22
TC (mg/dL)	84.61 ± 6.95	195.63 ± 12.31^*^	1 62.92 ± 23.50
TG (mg/dL)	92.17 ± 10.6	86.25 ± 9.58	1 100.15 ± 16.89
Liver			
TC (mg/g wet tissue)	0.89 ± 0.17	2.21 ± 0.33^*^	1.84 ± 0.31^#^
TG (mg/g wet tissue)	12.55 ± 3.58	20.36 ± 2.41^*^	15.56 ± 3.25^#^

^1^LFD, low-fat diet; HFD, 60% high-fat diet; CO-EtOAc, ethyl acetate fraction of Chinese olive; EAT, epididymal adipose tissue; TC, total cholesterol; TG, triacylglyceride.

^2^Each value is represented as mean ± SEM. (n = 6 per group, ^*^p < 0.05 versus LFD, ^#^*p* < 0.05 versus HFD).

^3^Details of the nutrient contents, feeding, and treatment period are given in the “Materials and Methods” section.

## Discussion

Epidemiological studies and relevant meta-analyses strongly suggest that long-term consumption of a diet rich in polyphenols offers protection against metabolic disorders such as type 2 diabetes, cardiovascular disease, and non-alcoholic fatty liver disease^45,46^. Many phenolic compounds have been identified in Chinese olive fruit, including gallic acid, methyl gallate, ethyl gallate, sinapic acid, brevifolin, brevifolin carboxylic acid, 3-*O*-galloyl quinic, ellagic acid, astragalin, hyperin, amentoflavone, and corilagin^47^. Using high Performance liquid chromatography (HPLC) analysis, we found that ellagic acid and gallic acid are the major ingredients in CO-EtOAc^48^. Both ellagic acid and gallic acid are dietary polyphenols with increasing attention due to their potent antioxidant activity and their marked effects on the prevention of oxidative stress^49^. Interactions between small dietary components and various biological macromolecules provide essential roles in the regulation of physiological functions via specific mechanism. Up to date, there is seldom systemic study on the interconnected networks of polyphenolic compounds in prevention of NAFLD under high fat diet administration. To our knowledge, this is the first study to investigate the effect of CO-EtOAc on hepatic damage caused by an HFD. We evaluated the protective effect of CO-EtOAc against hepatic lipid accumulation in HFD-treated animals and its regulation of genes and proteins involved in lipid metabolism.

We also used FL83B mouse hepatocytes as a cellular model to evaluate the protective effect of CO-EtOAc under high concentrations of oleic acid (OA, C18:1). OA is a product formed from the desaturation of stearic acid and is an essential end product of *de novo* fatty acid synthesis^50^. OA is abundant within the triglycerides of hepatocytes in both healthy subjects and patients with hepatic steatosis^51^. Additionally, OA has shown to be more steatotic than other FFAs^52,53^. The current study reveals that OA significantly increased lipid accumulation, ROS production, and lipid peroxidation in FL83B hepatocytes. However, CO-EtOAc significantly ameliorated the lipotoxic effects of fatty acid overloading in hepatocytes. To further characterize the CO-EtOAc-mediated hepatic lipid metabolism under high fat challenge, changes in AMPK activation were investigated. AMPK is a sensor that can switch cellular metabolism from anabolic to catabolic mode^54^. Our data demonstrated that CO-EtOAc is able to elevate AMPK phosphorylation and thereby attenuates hepatic lipid accumulation. We then used a CaMKKβ inhibitor STO-609 to validate whether CaMKKβ is involved in the CO-EtOAc-induced AMPK phosphorylation. The results showed that CaMKKβ was apparently involved in CO-EtOAc-mediated AMPK phosphorylation (Fig. S1A), while LKB1 was not (Fig. S1B). Several studies have also revealed that AMPK is sensitive to AMP/ATP ratio^55,56^. AMPK might be activated in response to the increased ratio of AMP/ATP. Our observation that CO-EtOAc treatment enhanced intracellular AMP/ATP ratio (Fig. S1C) raised the possibility that AMP/ATP ratio might be also another factor involved in CO-EtOAc-induced AMPK activation.

An enhanced expression of SREBP-1c has been observed in NAFLD^57^, however, the regulation of this transcription factor in fatty liver is not fully clarified. Mendez-Sanchez, N. *et al*. have demonstrated that an increase in fatty acid delivery to hepatocytes induces hepatic DNL via the overexpression of SREBP-1c^58^. SREBP-1c upregulates genes required for lipogenesis and fatty acid transport, including FAS, ACC1, and CD36^59^. Furthermore, hepatic FFA influx is strongly regulated by FATPs, including caveolins, CD36, and FABP^60^. Musso, G. *et al*. reported that the accumulation of fatty acids creates a diffusional gradient across the plasma membrane via CD36^61^. Moreover, FAS and ACC1 play an essential role in the synthesis of *de novo* fatty acids and TGs in the liver, and FAS inhibitors have been used as a potential treatment for obesity and liver-related disorders^62^. In the present study, we found that CO-EtOAc inhibited OA-mediated increases in the levels of mature SREBP-1c, CD36, and FAS proteins in FL83B cells. We thus consider that CO-EtOAc inhibits fatty acid biosynthesis and lipid accumulation in hepatocytes.

We also investigated the effect of CO-EtOAc on other lipid metabolic mediators, such as PPARα, CPT-1, and ACOX. PPARα is a transcription factor predominantly expressed in the liver and is an essential regulator of lipid metabolism. A number of studies have shown that PPARα modulates fatty acid uptake and β-oxidation^63–65^. Moreover, OA treatment significantly suppresses PPARα expression, thus induces lipid accumulation in hepatocytes^66,67^. In this study, we found that CO-EtOAc may increase PPARα expression in the OA-treated FL83B cells, which are thought to be involved in the increase in fatty acid oxidation. Impaired fatty acid oxidation leads to excess lipid accumulation and metabolic disorders. In contrast, increased fatty acid oxidation can aid the prevention of metabolic disorders such as obesity, dyslipidaemia, NAFLD, and NASH. Other studies have shown that supplementation with extra virgin olive oil may normalize the fall in the hepatic levels of n-3 LCPUFAs and subsequently reduce oxidative stress and liver steatosis in mice treated with HFD^68,69^. It seems to be a consensus that regulation of lipid metabolism and reduction of oxidative stress associate with the attenuation of liver steatosis. The results of our animal study also show that CO-EtOAc exhibits an inhibitory effect on hepatic lipid accumulation through regulation of diverse adipogenesis-related genes and proteins. Based on these findings, we believe that CO-EtOAc could be beneficial for prevention of the progression of NAFLD and NASH through its hepatoprotective mechanisms.

Overall, CO-EtOAc not only inhibited ROS production and TBARS levels in OA-treated FL83B cells, it decreased the protein expression levels of fatty acid transporters (CD36 and FABP) and lipogenic modulators, such as SREBP1c, FAS, and ACC1 both *in vitro* and *in vivo*. CO-EtOAc also upregulated mRNA of lipolysis gene *HSL* and lipid oxidation factors *PPARα*, *CPT-1*, and *ACOX*. We suggest that CO-EtOAc may activate lipid oxidation and inhibit lipogenesis at least partially through AMPK activation in hepatocytes. Moreover, CO-EtOAc is able to reduce body weight gain and hepatic lipid accumulation in mice challenged with an HFD. Our results strongly indicate that Chinese olive fruit may have a therapeutic role in the prevention of metabolic abnormalities in fatty liver disease.

## Methods

### Preparation of Chinese olive fruit extract

**Figure 7:**
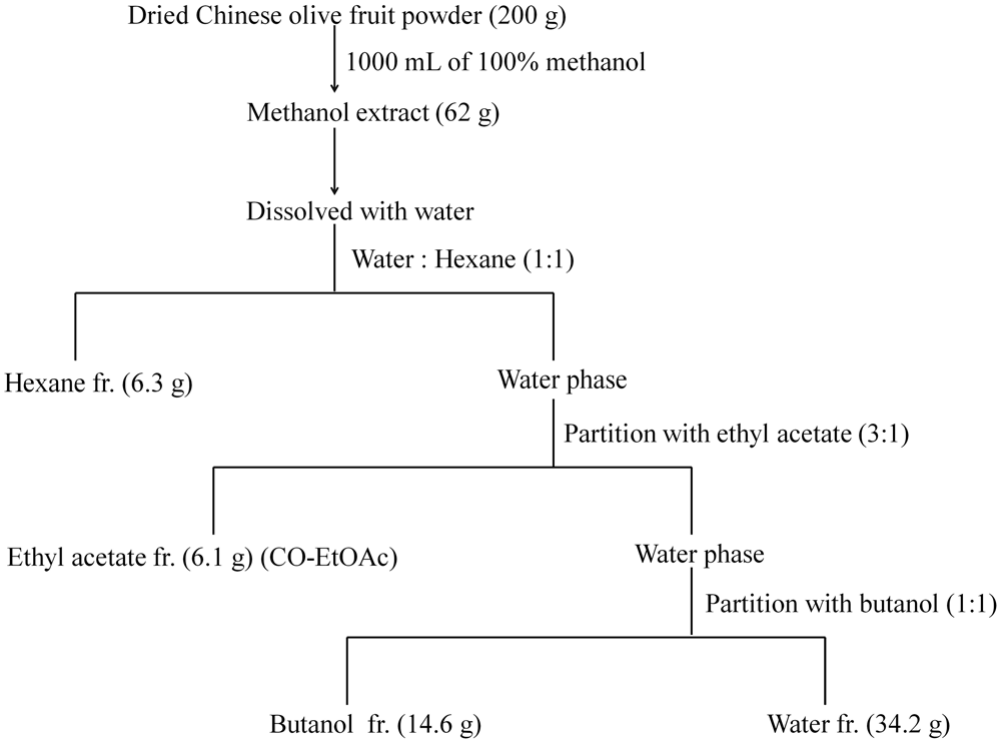
Flow chart of Chinese olive fruit extraction steps.

Chinese olive fruits were obtained from Baoshan Township, Hsinchu County, Taiwan. Whole fruits were dried and milled into a powder, 200 g of which was added to 100% methanol at room temperature for 3 h. The yield of methanol extract was 62 g, which was then dissolved in water and partitioned with n-hexane in a ratio of 1:1 (v/v). The water layer was further partitioned with ethyl acetate in a ratio of 3:1 (v/v). Finally, the water layer was partitioned with n-butanol in a ratio of 1:1 (v/v). The fractionation flow and solid content of each fraction are described in Fig. 7.

### Cell culture

FL83B cells were obtained from the Taiwan Bioresource Collection and Research Center and maintained in F12K medium (Sigma-Aldrich, St Louis, MO, USA) containing 10% fetal bovine serum and 1% penicillin/streptomycin at 37 °C in a humidified atmosphere with 5% CO_2_. CO-EtOAc was dissolved in 0.1% DMSO. Oleic acid (OA) (Sigma-Aldrich, St Louis, MO, USA) was dissolved in 100% ethanol and diluted in F12K medium containing 2% (w/v) BSA. Control groups were treated with the same concentration of DMSO to control the solvent effect.

### Oil red O staining

Intracellular lipid droplets were detected by oil red O (ORO) staining as described previously^70^. After treatment, cells were washed three times with iced PBS and fixed with 10% formaldehyde for 60 min. After fixation, cells were washed and stained with Oil Red O solution (stock solution, 5 mg/mL in isopropanol; working solution, 60% Oil Red O stock solution and 40% distilled water) for 30 min at room temperature. After staining, cells were washed with water to remove unbound dye. The stained lipid droplets within cells were visualized using a light microscope with a digital camera at 200X magnification and lipid accumulation was measured using a microplate reader at 490 nm.

### Cell viability assay

Cell viability was determined using an MTT (Sigma-Aldrich, St Louis, MO, USA) colorimetric assay as described previously^71^. On the first day, cells were plated at a density of 5000 cells per 96-well plate. On the second day, cells were incubated with 600 μM OA in the presence or absence of different concentrations of CO-EtOAc (50–400 μg/mL). After 24 h, the cells were treated with 0.5 mg/mL MTT solution and then incubated for an additional 3 h at 37 °C. The insoluble formazan products were dissolved in DMSO and the absorbance read using an ELISA reader at 595 nm.

### Reactive oxygen species (ROS) production assay

Intracellular ROS concentrations were measured using 2′,7′-dichlorodihydrofluorescein diacetate (H_2_DCFDA) (Sigma-Aldrich, St Louis, MO, USA), as described previously with slight modification^72^. Briefly, cells were incubated with H_2_DCFDA at a final concentration of 10 μM in medium for 30 min at 37 °C followed by our experimental treatments. After 30 min, fluorescence was measured at an excitation wavelength of 485 nm and an emission wavelength of 535 nm using a fluorescence reader.

### Thiobarbituric acid-reactive substance (TBARS) assay

Lipid peroxidation was estimated by determining the production of TBARS and was expressed as malondialdehyde (MDA) equivalents^73^. Briefly, cells were reacted with 20% trichloroacetic acid and 1% thiobarbituric acid. The reaction mixture was incubated at 90 °C for 20 min and stopped on ice. After cooling to room temperature, TBARS were extracted with 0.5 mL n-butanol and separated by centrifugation at 3000 × g for 5 min. The absorbance of TBARS was determined at 535 nm.

### RNA extraction and qRT-PCR

Total RNA was isolated using Trizol Reagent (Roche; Basel, Switzerland) according to manufacturer’s protocol and quantified by spectrophotometry at 260 nm. 2 μg of RNA was reverse-transcribed into single-strand cDNA with oligo dT and SMART MMLV Reverse Transcriptase (Clontech; Palo Alto, California, USA) according to the manufacturer’s instructions. Quantitative reverse transcription polymerase chain reaction (qRT-PCR) analyses were performed on aliquots of cDNA preparations to detect gene expression using StepOnePlus™ Real-Time PCR Systems (Thermo Fisher Scientific. Waltham, Massachusetts, USA). Reactions were carried out in 10 μL volumes containing: 5 μL 2 × KAPA SYBR^®^FAST qPCR Master Mix (2X) ABI Prim™, 0.2 μL forward and 0.2 μL reverse primers, 2.6 μL water, and 2 μL cDNA. 40 amplification cycles were performed for denaturation at 94 °C for 60 s, annealing at 63 °C for 60 s and extension at 74 °C for 3.5 min. The primers used to amplify each target gene are described in Supplemental Table S1. PCR primers used in this study were purchased from Genomics BioSci & Tech, Taiwan. mRNA levels were analysed using 2^−ΔΔCT^ method. Relative mRNA expression levels were calculated as a ratio of each transcript relative to the housekeeping gene, r18S.

### Isolation of nuclear extracts

FL83B cells from five 15-cm plates were washed twice with PBS and collected by centrifugation at 1,000 × g for 3 min. Pelleted cells added hypotonic buffer (10 mM HEPES pH 7.0, 10 mM KCl, 1 mM magnesium acetate, 1 mM DTT, 10% glycerol, 1 mM phenylmethylsulfonyl fluoride, 0.5% NP-40) and protease inhibitor cocktail (Roche Diagnostics GmbH, Mannheim, Germany); the cell suspension was kept on ice for 5 min followed by centrifugation at 1,200 × g for 5 min at 4 °C (the supernatants is cytosolic protein). Pelleted cells added hypertonic buffer (10 mM HEPES pH 7.0, 0.5 M KCl, 1 mM magnesium acetate, 1 mM DTT, 10% glycerol, 1 mM phenylmethylsulfonyl fluoride, 0.5% NP-40, 5 mM EDTA) and protease inhibitor cocktail; the cell suspension was kept on ice for 30 min (vortex every 5 min) followed by centrifugation at 1,200 × g for 5 min at 4 °C; the supernatant containing nuclear proteins was collected.

### Western blot analysis

Cell lysates were prepared using RIPA Lysis Buffer (Merck Millipore, Rahway, NJ, USA) containing protease inhibitor cocktail (Roche Diagnostics GmbH, Mannheim, Germany) and phosphatase inhibitor cocktail I and II. After centrifugation at 12,000 × g for 12 min, the supernatant was collected. The concentrations of proteins from cell lysates were determined by Bradford assay with BSA as a standard. Equal amounts of proteins (30 μg) were subjected to 8–15% sodium dodecyl sulphate-polyacrylamide gel electrophoresis (SDS-PAGE) and then transferred to nitrocellulose membranes (Pall, Glen Cove, NY). The immunoblots were blocked with 5% nonfat milk in PBST (0.26 M KH_2_PO_4,_ 0.1 M Na_2_HPO_4,_ 1.45 M NaCl, 0.1% Tween-20) for 1 h at room temperature, and blotted membranes were then incubated with first antibodies against phospho-AMPKα (Thr-172), phospho-ACC1 (Ser-79), total AMPKα, and total ACC1 (Cell Signaling Technology, Beverly, MA, USA), CPT-1, PPARα, FAS, β-actin (Abcam, Cambridge, UK), SREBP-1c (Thermo Scientific, Rockford, IL, USA), LKB1, and phosphor-LKB1 (Santa Cruz, CA, USA). overnight at 4 °C. The membrane was washed with PBST three times, followed by incubation with goat anti-rabbit or mouse IgG horseradish peroxidase (HP)-conjugated secondary antibodies (Thermo Scientific, Rockford, IL, USA), and the expression of proteins was detected with enhanced chemiluminescence reagent (ECL, PerkinElmer, Boston, MA, USA). The intensity of protein for each band was quantified by densitometry using Image Quant software (Molecular Dynamics, Sunnyvale, CA).

### HPLC quantification of AMP and ATP

Intracellular ATP and AMP contents were measured by HPLC^74^. In brief, FL83B cells grown in the presence or absence of CO-EtOAc were washed twice with cold phosphate-buffered saline and immediately centrifuged for 2 min at 1000 × g (4 °C). Cell pellets were lysed in 150 μl of perchloric acid (4% v/v) and the lysates were then neutralized with 2 M KOH/0.3 M 3-(N-morpholino)propanesulfonic acid (MOPS) (Sigma-Aldrich, St Louis, MO, USA). After centrifuging at 13,000 × g for 10 min, the supernatant was analyzed by HPLC (HPLC-Pro Star from Varian, Walnut Creek, CA) with a Spherisorb column (ODS II, 5 mm, 0.46 × 25 cm, Z22.697-1, Sigma-Aldrich, St Louis, MO, USA) at a flow rate of 1.0 ml/min. The mobile phase used was an isocratic mixture of 20 mM KH_2_PO_4_ and 3.5 mM K_2_HPO_4_-3H_2_O at pH 6.1. The adenine nucleotides were analyzed spectrophotometrically at 254 nm. Each elution peak was compared with AMP, ADP, and ATP standards (Sigma-Aldrich, St Louis, MO, USA) to confirm its identity. The order of eluted nucleotides was ATP, ADP, and AMP.

### Animals and diets

Four-week-old male C57BL/6 mice (initial weights 16 ± 1.5 g) were purchased from the National Laboratory Animal Center, Taiwan, and maintained in National Yang-Ming University. Animals were kept under a controlled temperature of 23 ± 2 °C and 55 ± 5% relative humidity with a 12 h light/dark cycle. After two weeks of acclimatization, mice were randomly assigned into three groups including (A) low-fat diet (LFD), (B) 60% high-fat diet (HFD), (C) 60% HFD + CO-EtOAc (n = 6 for each group). All study protocols were approved by the Institutional Animal Care and Use Committee of National Yang-Ming University. In addition, all experiments were performed in accordance with relevant guidelines and regulations. Mice in the LFD group were allowed free access to a basal diet (#58Y2 Test Diet, St. Louis, MO, USA), providing 18.0% of energy as protein (16.9 g/100 g), 10.2% of energy as fat (4.3 g/100 g), and 71.8% of energy as carbohydrate (67.4 g/100 g). Mice in the HFD were provided with a commercial diet (*TD.06414* -Adjusted Calories Diet-60/Fat, Harlan laboratories, Madison, USA) consisting of 60% fat. Of the CO-EtOAc group, mice were administered 150 mg/kg body weight of CO-EtOAc in 0.5% carboxymethyl cellulose (v/v) intragastrically for four weeks. LFD and HFD group animals were also gavaged with 0.5% carboxymethyl cellulose. During the 21-week experimental period, mice were free to access food and water. Animals were sacrificed by CO_2_ anaesthesia after fasting for 12 h, and serum was collected for biochemical analysis. The liver was dissected out and stored at −80 °C for further experiments.

### Biochemical analysis

Blood glucose was determined using a glucose analyser (Eumed Biotechnology Co., Ltd., Hsinchu, Taiwan). Serum levels of insulin were determined using commercial kits according to the manufacturer’s instructions (Mercodia AB, Uppsala, Sweden). Serum levels of TG and TC were assayed using a colorimetric assay kit according to the manufacturer’s instructions (Cayman, MI, USA).

### Hepatic triglyceride and cholesterol determination

Lipids were extracted from liver samples following the modified method described by Folch *et al*.^75^. Briefly, total lipids were extracted from the liver samples by homogenizing the tissues with 8:4:3 chloroform/methanol/0.9% NaCl (v/v) to a final dilution of 20 times the original volume of the tissue sample. The organic layer was then separated, evaporated, and reconstituted in chloroform. The levels of TG and TC were measured using a colorimetric assay kit (Cayman, MI, USA) and normalized against the weight of the extracted liver.

### Histological analysis

The right medial lobes of mouse liver were fixed in 10% (v/v) formalin solution at room temperature for 48 h. The liver tissue was subjected to the processes of washing, dehydration, clearing, and infiltration, and was embedded in 100% paraffin wax for 3 h. Each sample was sliced into a section of 4–5 μm in thickness, followed by haematoxylin and eosin (H&E) staining. The slides were then examined under a light microscope equipped with a digital camera under 100X magnification, and at least 10 areas of each slide were observed.

### Statistical analysis

All data are expressed as mean ± SEM of at least three independent experiments. Evaluations of normality data distribution was performed using the Shapiro Wilk test. Assessment of the statistical significance between control and experimental groups were made using one-way ANOVA with the Tukey’s method as a *post*-*hoc* test. *P* < 0.05 was considered statistically significant.

## Electronic supplementary material

Supplementary Information
