# Supplementary material for: Chinese olive extract ameliorates hepatic lipid accumulation in vitro and in vivo by regulating lipid metabolism
Source: Sci Rep. 2018 Jan 18;8:1057. doi: 10.1038/s41598-018-19553-1 (PMC5773498; doi:10.1038/s41598-018-19553-1)
Supplement: Supplementary file 1 — Supplementary Information [file 41598_2018_19553_MOESM1_ESM.pdf]

Supplementary Information

Chinese olive extract ameliorates hepatic lipid accumulation *in vitro* and *in vivo* by regulating lipid metabolism

Yu-Te Yeh[1], Yan-Yu Cho[2], Shu-Chen Hsieh[1,*], An-Na Chiang[2,*]

[1]Institute of Food Science and Technology, National Taiwan University, Taipei, Taiwan.

[2]Institute of Biochemistry and Molecular Biology, National Yang-Ming University, Taipei, Taiwan.

Table S1. Primer sequences used for qRT-PCR analysis

Gene	Forward primer	Reverse primer
CD36	GCTTGCAACTGTCAGCACAT	GCCTTGCTGTAGCCAAGAAC
FAS	CTGAGATCCCAGCACTTCTTGA	GCCTCCGAAGCCAAATGAGTGA
SREBP-1c	AGGTGTATTTGCTGGCTTGGT	AGAGATGACTAGGGAACTGTGTGT
ACC1	GGCTGGGCTTGTATAAGT	GATAATATGGCAATCTCAGT
PPARα	CCTGCTTCCTGCCACTTG	GTTCACCCTGATTCCTGA
CPT-1	ACATCCCTAAGCAGTGCCAG	TCGTCCGGCACTTCTTGATC
FABP	CTGTGGAAAGGAAGCCTCGTT	CTCAAAGTTCTCCTGGCTCTGC
ACOX	ACCAACTGTCACACTAACATATCA	GGCATGTAACCCGTAGCACT
ATGL	CTTGAGCAGCTAGAACAATG	GGACACCTCAATAATGTTGGC
HSL	GCTGGAGGAGTGTTTTTTTGC	AGTTGAACCAAGCAGGTCACA
MGL	TCGGAACAAGTCGGAGGT	TCAGCAGCTGTATGCCAAAG
r18S	TATTCCCATGACCCGCC	GTGAGGTTTCCCGTGTT

A

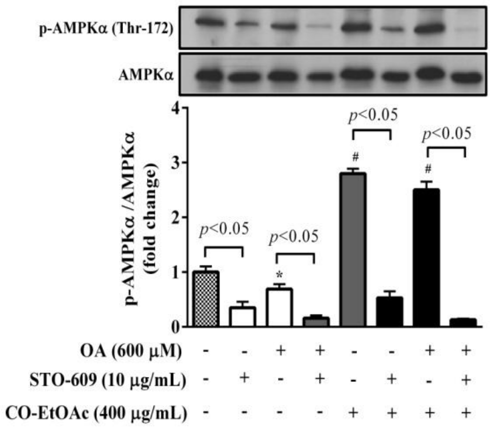

p-AMPKα (Thr-172)
AMPKα
p-AMPKα /AMPKα (fold change)
p<0.05
p<0.05
p<0.05
p<0.05
OA (600 μM)
STO-609 (10 μg/mL)
CO-EtOAc (400 μg/mL)

B

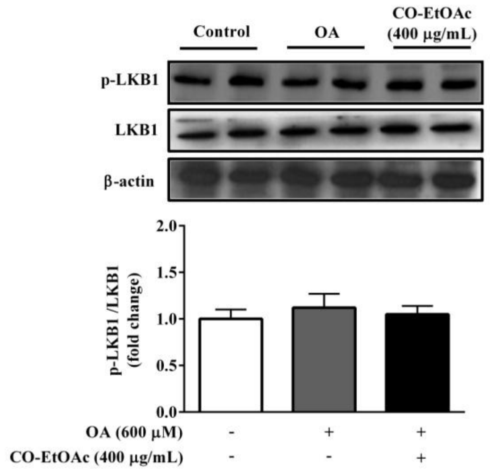

Control
OA
CO-EtOAc (400 μg/mL)
p-LKB1
LKB1
β-actin
p-LKB1 /LKB1 (fold change)
OA (600 μM)
CO-EtOAc (400 μg/mL)

C

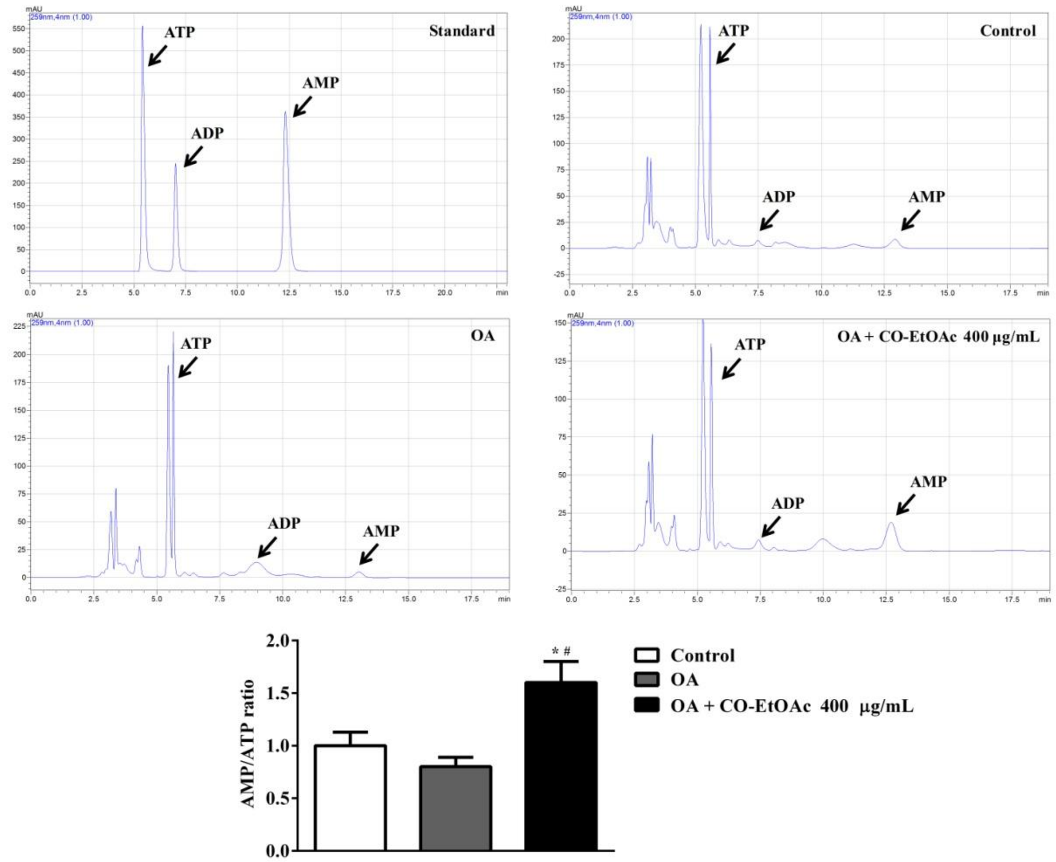

Standard
Control
OA
OA + CO-EtOAc 400 μg/mL
ATP
ADP
AMP
AMP/ATP ratio
Control
OA
OA + CO-EtOAc 400 μg/mL

Figure S1. The involvement of CaMKKβ, LKB1, and AMP/ATP ratio in CO-EtOAc-induced AMPK activation. Cells were treatment with 600 μM OA for 3 h and then treated with 400 μg/mL of CO-EtOAc for another 21 h after pre-incubation. The levels of protein expression of (A) phosphorylated/total AMPKα in the presence or absence of 10 μg/mL STO-609 (a CaMKKβ inhibitor) for 1 h before CO-EtOAc treatment and (B) phosphorylated/total LKB1 were measured by western blot analysis. (C) Intracellular ATP, ADP, and AMP contents were analyzed using HPLC. The position of ATP, ADP and AMP are indicated by arrows. The histogram shows the ratios of AMP:ATP for each group. All results are expressed as the mean ± SEM of three independent experiments. *$p < 0.05$ versus the DMSO control group; $^{\#}$ $p < 0.05$ versus the OA group.
